# Mitogenomic Phylogenetics of Fin Whales (*Balaenoptera physalus spp.*): Genetic Evidence for Revision of Subspecies

**DOI:** 10.1371/journal.pone.0063396

**Published:** 2013-05-17

**Authors:** Frederick I. Archer, Phillip A. Morin, Brittany L. Hancock-Hanser, Kelly M. Robertson, Matthew S. Leslie, Martine Bérubé, Simone Panigada, Barbara L. Taylor

**Affiliations:** 1 Southwest Fisheries Science Center, National Marine Fisheries Service, La Jolla, California, United States of America; 2 Scripps Institution of Oceanography, University of California San Diego, La Jolla, California, United States of America; 3 Centre for Ecological and Evolutionary Studies, University of Groningen, Groningen, The Netherlands; 4 Tethys Research Institute, Milano, Italy; BiK-F Biodiversity and Climate Research Center, Germany

## Abstract

There are three described subspecies of fin whales (*Balaenoptera physalus*): *B. p. physalus* Linnaeus, 1758 in the Northern Hemisphere, *B. p. quoyi* Fischer, 1829 in the Southern Hemisphere, and a recently described pygmy form, *B. p. patachonica* Burmeister, 1865. The discrete distribution in the North Pacific and North Atlantic raises the question of whether a single Northern Hemisphere subspecies is valid. We assess phylogenetic patterns using ∼16 K base pairs of the complete mitogenome for 154 fin whales from the North Pacific, North Atlantic - including the Mediterranean Sea - and Southern Hemisphere. A Bayesian tree of the resulting 136 haplotypes revealed several well-supported clades representing each ocean basin, with no haplotypes shared among ocean basins. The North Atlantic haplotypes (n = 12) form a sister clade to those from the Southern Hemisphere (n = 42). The estimated time to most recent common ancestor (TMRCA) for this Atlantic/Southern Hemisphere clade and 81 of the 97 samples from the North Pacific was approximately 2 Ma. 14 of the remaining North Pacific samples formed a well-supported clade within the Southern Hemisphere. The TMRCA for this node suggests that at least one female from the Southern Hemisphere immigrated to the North Pacific approximately 0.37 Ma. These results provide strong evidence that North Pacific and North Atlantic fin whales should not be considered the same subspecies, and suggest the need for revision of the global taxonomy of the species.

## Introduction

Fin whales (*Balaenoptera physalus* Linnaeus, 1758) are distributed across the temperate to subpolar waters of the world. The Society of Marine Mammalogy currently recognizes three subspecies: *B. p. physalus* Linnaeus, 1758 in the Northern Hemisphere, *B. p. quoyi* Fischer, 1829 in the Southern Hemisphere, and the pygmy fin whale, *B. p. patachonica* Burmeister, 1865 [1]. The distinction between Northern and Southern Hemisphere fin whales was first proposed in a comparative study of the osteology of Bryde’s whales (*Balaenoptera edeni* Anderson, 1879) in which Lönnberg [2] noted differences in the vertebral characteristics of fin whales in the two hemispheres, leading to the suggestion that Southern Hemisphere whales were a different subspecies, bearing the name *B. p. quoyi*. Tomilin [3] independently noted both the larger mean and maximum body sizes of Southern Hemisphere whales, also suggesting that subspecific status was warranted under the same name. These differences were later verified by morphological examinations of a larger series of specimens in a study of body measurements and organ weights of fin whales from the North Atlantic and Antarctica [4], which found that Antarctic fin whales have a greater percentage of blubber weight than those caught off of Iceland while having similar muscle weights, making the Icelandic whales appear leaner. The maximum body length of fin whales in the Antarctic (>23 m) was about 3–4 m greater than those in the Northern Hemisphere [4], [5].

The establishment and recognition of the Southern Hemisphere *B. p. quoyi* automatically placed all Northern Hemisphere fin whales within the nominate subspecies *B. p. physalus*. Differentiation between the hemispheres is a pattern mirrored in many cetaceans [6], [7] and is well supported. In contrast, the default condition that all Northern Hemisphere fin whales belong to the same subspecies (*B. p. physalus*) has not been evaluated and is unlikely given their disjunct distribution in the North Atlantic and North Pacific.

Recently, Clarke [8] has presented evidence that more than one form of fin whale may exist in the Southern Hemisphere in his description of the pygmy fin whale *B. p. patachonica* Burmeister, 1865 [8]. The form is described as small (approximately 18–24 m) and dark with black baleen [9]–[11]. The type specimen was collected from a stranding at the mouth of the Rio de la Plata, Argentina [12] at approximately 36°S, and Clarke [8] suggests that they do not extend further south than approximately 55°S.

Recent genetic and acoustic studies on this species have focused on population-level differentiation within ocean basins in the Northern Hemisphere, although some limited comparisons between North Atlantic and North Pacific populations have been conducted. Bérubé et al [13] found no shared mitochondrial DNA (mtDNA) haplotypes between fin whales sampled in the Gulf of California and those sampled in the strata containing the North Atlantic and Mediterranean; the average *F_st_* value for six microsatellite loci was 0.51 [14], which is several times larger than the mean *F_st_* of 0.12 for comparisons between North Atlantic populations and the Mediterranean Sea. Hatch and Clark [15] found a significant correlation between measures of mtDNA and microsatellite differentiation and geographic distance among fin whales from various sites in the North Pacific and North Atlantic, but no correlation using paternally inherited DNA from the y-chromosome (yDNA), which the authors interpreted as suggesting some degree of male-mediated dispersal. However, because their analyses were not hierarchically stratified by geography, they did not address the question of yDNA differentiation between ocean basins. In other words, most of the correlation they present could be due to within-ocean-basin comparisons. Additionally, while not fully diagnostic, Hatch and Clark [15] also found that 82% of singing whales from the North Pacific, North Atlantic, and Mediterranean Sea could be correctly classified to the region within the ocean basin from which they were sampled based on components of their calls. Those that were misclassified were most often misclassified to a region within the same ocean basin, indicating ocean-basin-specific call components.

It is very unlikely that significant gene flow has occurred between North Pacific and North Atlantic fin whales at least since the closing of the Isthmus of Panama around 4 Ma [16], [17]. During the feeding season, North Pacific fin whales are not known to extend past the Bering Sea into the Chukchi Sea farther north than approximately 70**°**N [18]. In the North Atlantic, they occur in the Norwegian Sea and have been detected up to 80**°**N in the Greenland Sea and approximately 75**°**N in the Barents Sea [19], [20]. On the western side of Greenland, they have been detected to about 70**°**N in Davis Strait [21]. During research cruises in the eastern tropical Pacific from August to December, they are rarely encountered south of the Baja Peninsula, Mexico [22], and are extremely rare if not entirely absent in the western Caribbean Sea or Gulf of Mexico [23]–[25].

We present the first study of the phylogenetic relationship of fin whales from three of the primary ocean basins in which they occur: the North Atlantic, North Pacific, and Southern Ocean. Historically, phylogeographic analyses of large whales have proven to be a difficult endeavor. This is a result of their overall body sizes and widespread distribution, which makes it difficult to accumulate, archive, and examine a sufficient number of osteological specimens from across their range. The rapid development of new molecular techniques, such as Next Generation Sequencing (NGS) [26], [27], as well as computer-intensive analytical methods, such as Bayesian phylogenetics [28] has offset some of those problems by extracting more information from the available soft tissue samples, thus improving our understanding of patterns of divergence at multiple taxonomic levels [29]–[31]. Here we examine the phylogenetic relationship as well as the degree and timing of divergence of fin whales between each ocean basin using the complete mitochondrial DNA sequence generated using NGS. We also examine the geographical distribution of clades based on mitogenome sequences within a larger dataset of control region sequences generated with standard Sanger sequencing methods.

## Methods

### Ethics Statement

Procedures for ensuring animal welfare during biopsy sampling were approved as part of the Scientific Research permits issued by the National Marine Fisheries Service under the authority of the Marine Mammal Protection Act of 1972 (16 U.S.C. 1361 et seq), the regulations governing the taking and importing of marine mammals (MMPA) (50 CPR part 216), the Endangered Species Act of 1973 (ESA) (16 U.S.C. 1531 et seq.), and the regulations governing endangered fish and wildlife permits (50 CFR parts 222–226). Biopsies were taken under NMFS permit numbers 779–1339, 779–1663, 14097, 774–1437, 774–1714, 1026/689424, and 873 issued to the National Marine Fisheries Service Southeast Fisheries Science Center and Southwest Fisheries Science Center. The samples originating from outside US jurisdiction were imported under CITES Import permit numbers US774223 and US689420, and under CITES Certificate of Scientific Exchange #690343. CITES permits are issued by the U.S. Fish & Wildlife Service. The Southwest Fisheries Science Center is a Registered Scientific Institution under CITES (US052).

### Samples

**Figure 1:**
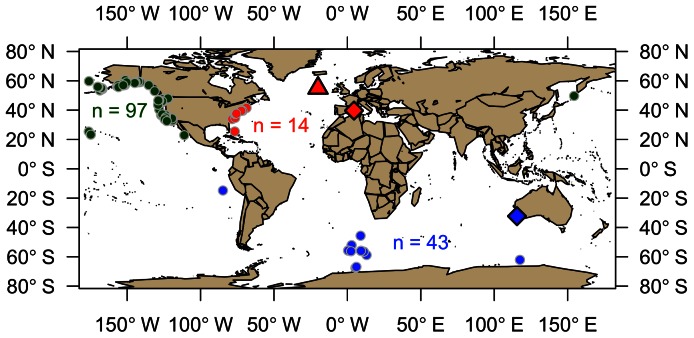
Location of fin whale mitogenome samples (n = 154). Each small circle represents a single sample. Larger symbols are: red triangle = reference sequence from Arnason et al (1998), red diamond = Mediterranean Sea samples (n = 5), blue diamond = stranded western Australia samples (n = 2).

A total of 435 fin whale samples were collected from the North Pacific, North Atlantic, Mediterranean Sea, and Southern Ocean (Figure 1). Samples from the North Pacific, North Atlantic, and Mediterranean Sea would currently be classified as Northern Hemisphere fin whales (*Balaenoptera physalus physalus*), while those from the Southern Ocean represent Southern Hemisphere fin whales (*B. p. quoyi*). We were unable to obtain any samples that could be positively identified as pygmy fin whales (*B. p. patachonica*) for this study. Most samples are from biopsies of living whales taken at sea, but a few North Pacific samples were from stranded individuals. To ensure that our mitogenome dataset represented the full range of mitochondrial diversity in each ocean basin, we selected a subset of these samples based on mitochondrial control region (CR) haplotypes previously generated for another project via Sanger-sequencing as described below.

### Control Region Sanger-Sequencing

For 430 samples, we sequenced the first 412 base pairs of the hypervariable mtDNA conrol region (CR). Genomic DNA was extracted using several standard methods including a sodium chloride protein precipitation (modified from Miller et al [32]), a silica-based filter purification (DNeasy kit; Qiagen, Valenica, CA, USA), or a silica-based robotic extraction using QIAxtractor DX reagents (Qiagen, Valenica, CA, USA). PCR reactions using primers TRO (5′-CCTCCCTAAGACTCAAGG-3′; developed at SWFSC) and D (5′-CCTGAAGTAAGAACCAGATG-3′ [33]) were performed in 25 µl volumes using 1 µL (approximately 5–25 ng) genomic DNA, 1× PCR buffer [10 mm Tris–HCl (pH 8.3), 50 mm KCl, 1.5 mm MgCl_2_], 0.3 µm of each primer, 200 µm of each dNTP and 0.5 units of *Taq* DNA polymerase. The PCR thermal profile consisted of an initial denature at 94°C for 2.5 min, followed by 35 cycles of 94°C for 45 sec, 56°C for 1 min, and 72°C for 1.5 min, then a final extension at 72°C for 5 min. Sanger-sequencing of the PCR product in both directions was performed using the ABI 3100, 3130XL, and 3730 Automated Sequencers (Applied Biosystems Inc., Foster City, CA). All sequences were aligned using Sequencher v4.1 software (Gene Codes Corp., 2000; Ann Arbor, MI). To test for errors in sequencing, a random 10% replication of all samples was completed. If a discrepancy was found, the sample was re-sequenced from extracted DNA. Unresolved discrepancies and all rare haplotypes were re-extracted from tissue and re-sequenced for haplotype confirmation.

### Mitogenome Next Generation Sequencing

We selected 148 of these Sanger-sequenced samples and an additional five samples from the Mediterranean Sea for Next Generation Sequencing (NGS) of the entire mitogenome. These 153 samples were selected to ensure that all CR haplotypes from each ocean basin were represented in the mitogenome dataset and all geographic regions within an ocean basin were represented. Mitogenome library preparation closely followed a modified capture array method [34] with a few modifications [35]. The capture arrays had five copies of each mtDNA probe sequence, and each array had three base pairs between the beginning of each 60 bp probe tiled across the mitochondrial sequences. The published fin whale mitogenome (Genbank Accession NC-001321 [36]) was used for design of capture probes using the web-based software eArray (Agilent Technologies, Inc., Santa Clara, CA, USA). The library preparation protocol included DNA fragmentation, blunt-end repair of the sheared DNA, ligation of adaptors, and individual labeling of the libraries via PCR amplification with indexed primers. The indexed libraries were then quantified and pooled in equimolar amounts and hybridized to the capture arrays to enrich the pooled library for the mitochondrial genome.

### Mitogenome Assembly

Consensus sequences were generated from mitogenome sequence reads using a custom pipeline written by FIA (available at the Dryad data repository, http://dx.doi.org/doi:10.5016/dryad.cv35b) in R version 2.15.0 [37]. Reads were first assembled to the reference fin whale sequence with the program BWA [38]. The *mpileup* module in SAMTOOLS [39] was used to convert the resulting BAM-format alignment file into a “pileup” text format that lists the base composition across reads at each site in the reference sequence. This text file was then parsed by custom R code to create the consensus sequence for each individual, using the following rules. If a given site had fewer than three reads, an “N” was placed in the consensus. If the coverage was between three and five, and all reads contained the same nucleotide, then that nucleotide was used in the consensus; otherwise, the consensus received an “N” for that site. If coverage was greater than five, then the nucleotide that occurred in 70% or more of the reads was used in the consensus. If no nucleotide frequency exceeded 70%, then an “N” was inserted. All mitogenome sequences (fin whale plus outgroup species) were initially aligned with MAFFT [40] followed by a refinement of alignments by eye.

We compared the first 412 base pairs of the control region in the NGS sequences with those from the Sanger-sequenced dataset for all but the five Mediterranean samples and the reference sequence. If the NGS sequence had an “N” at a site or was different from the base call in the Sanger-sequence, and the site had a strong, high-quality peak in the Sanger-sequence chromatogram, then it was replaced by the Sanger-sequence base pair. These Sanger-supplemented NGS sequences were then used in the phylogenetic analyses.

### Phylogenetic Analysis

We compiled sequences from five humpback whales (*Megaptera novaengliae*) as the outgroup to fin whales given their sister species relationships in several studies [41]–[43]. The full mitogenome sequence was available for one humpback sample (Genbank Accession # NC006927 [43]). The remaining four were partial sequences composed of only the coding genes (Genbank Accession #s FJ90425, Carraher et al unpublished data; GQ353254– GQ343292 representing individual coding regions from samples GOM9049, GOM9084, and SEA87041 [41]). With these sequences, we compiled two datasets, one composed of the complete 16,423 base pairs of the mitogenome with just the single humpback outgroup, and the other composed of 11,406 base pairs of the protein coding genes, using all five humpback samples (referred to below as “*mito*” and “*cds*” datasets respectively).

We estimated phylogenetic relationships and divergence times for both datasets using BEAST v1.7 [28]. In both the *mito* and *cds* datasets, all fin whale sequences were constrained to be monophyletic. The five humpback whale sequences were also constrained to be monophyletic in the *cds* dataset. Based on the results of an analysis with jModelTest [44], we selected the HKY+G substitution model with 4 substitution categories for both datasets. Models were run using a strict molecular clock for which we set a prior distribution on the mean substitution rate to *Uniform* (1e-5, 1e-2) based on the results of preliminary runs and published estimates of substitution rates in Cetacea [30], [41], [43], [45], [46]. A Yule speciation tree model was used and initialized with an UPGMA tree. The prior on the tree root was set as *Normal* (15.8, 2.8), corresponding to the estimate of time since most recent common ancestor (TMRCA) between fin and humpback whales [43]. A total of 10,000,000 MCMC iterations were run, with every 1,000^th^ iteration saved to create the posterior sample. Convergence and sufficient mixing of the posterior samples were evaluated by examination of the effective sample sizes (ESSs) and sampling traces for each parameter using Tracer v1.5 [47].

Supplemental material, including sample numbers, collection details, and GenBank accession numbers for each haplotype, parameters for mitogenome assembly, BEAST input files, full Bayesian posterior sample, and annotated trees are available at the Dryad data repository (http://dx.doi.org/10.5061/dryad.084g8).

## Results

There were a total of 103 CR haplotypes in the Sanger-sequenced data set (Table 1). Haplotypic diversity was high both within ocean basins as well as across all samples. The minimum diversity within an ocean basin was 0.828 for the North Atlantic, which also had the fewest samples. There were no shared haplotypes among ocean basins. There were two fixed differences between the North Atlantic and North Pacific (sites 181 and 198), and one between the North Atlantic and Southern Hemisphere sequences (site 198).

**Table 1:** Number of samples, haplotypes, and sequence diversity for each sequence dataset.

	Samples	Haplotypes	Variable Sites	Haplotypic Diversity
**Sanger CR**				
North Pacific	346	50	36	0.935
North Atlantic	28	12	16	0.828
Southern Hemisphere	48	41	36	0.993
Total	422	103	55	0.955
**Mitogenome CR**				
North Pacific	97	49	36	0.980
North Atlantic	8	8	14	1
Southern Hemisphere	43	41	36	0.997
Total	148	98	54	0.991
**Mitogenome**				
North Pacific	97	82	501	0.996
North Atlantic	14	12	97	0.967
Southern Hemisphere	43	42	438	0.999
Total	154	136	925	0.998

“Sanger CR” is the 412 bp of the control region generated from Sanger sequences. “Mitogenome CR” is the corresponding 412 bp of the control region from the NGS mitogenome sequence, and “Mitogenome” is the 16.4 Kbp full mitochondrial sequence generated from NGS reads. Note that the North Atlantic Mitogenome CR set does not include the 5 Mediterranean samples and the reference sequence from Arnason et al. (1991) for which no comparable Sanger sequences were generated.

Alignment of the mitogenome reads to the reference produced high quality consensus sequences 16,401 bp in length for most samples. The median number of reads aligning to the reference for each sample was 23,608. The median coverage (the number of reads aligning to a given site on the reference) per sample was 136. Of the 153 mitogenomes, 142 had at least one read covering every site. Of the remaining 11 sequences, the maximum number of sites with no coverage was 36. After alignment and base calling, approximately 33% of the consensus sequences had no missing bases. The median number of missing bases in a sequence was one, with 94% of the sequences having fewer than ten missing bases. There was no significant clustering of missing data within any particular region of the mitogenome, either across all samples or within samples from a given ocean basin. As expected, diversity in the mitogenome dataset was higher than in the Sanger control region sequences (Table 1). None of the 136 mitogenome haplotypes were shared among ocean basins.

**Figure 2:**
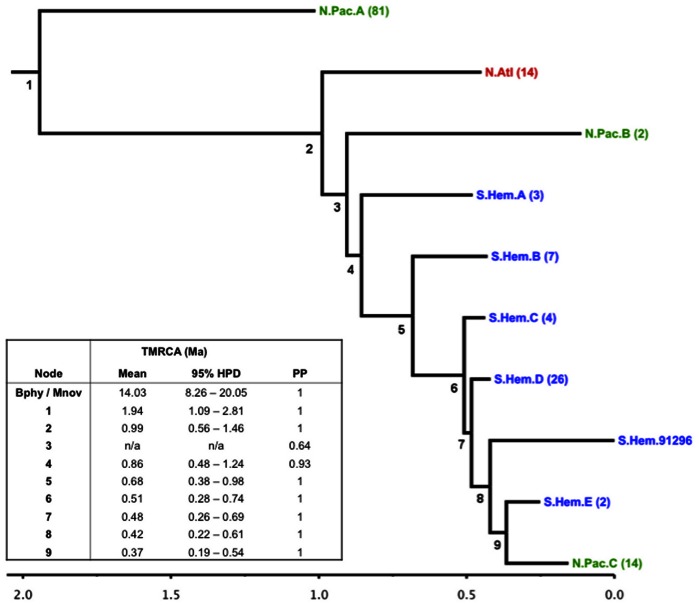
Summary of Bayesian fin whale phylogenetic tree using full mitogenome sequences (*mito* dataset). The root leads to divergence of fin whales from humpbacks. Branches with two or more samples in the same ocean basin have been collapsed. Numbers in parentheses are number of samples at each branch tip, except for the single Southern Hemisphere samples in clade 8 (SWFSC Lab ID 91296). Scale at bottom is node age in millions of years. Time to Most Recent Common Ancestor (TMRCA) estimates, 95% Highest Posterior Density (HPD), and posterior probabilities (PP) of each numbered node are given in the inset table. TMRCA values not reported for nodes with PP<0.9. The full annotated tree is available at the Dryad data repository, http://dx.doi.org/10.5061/dryad.084g8.

**Figure 3:**
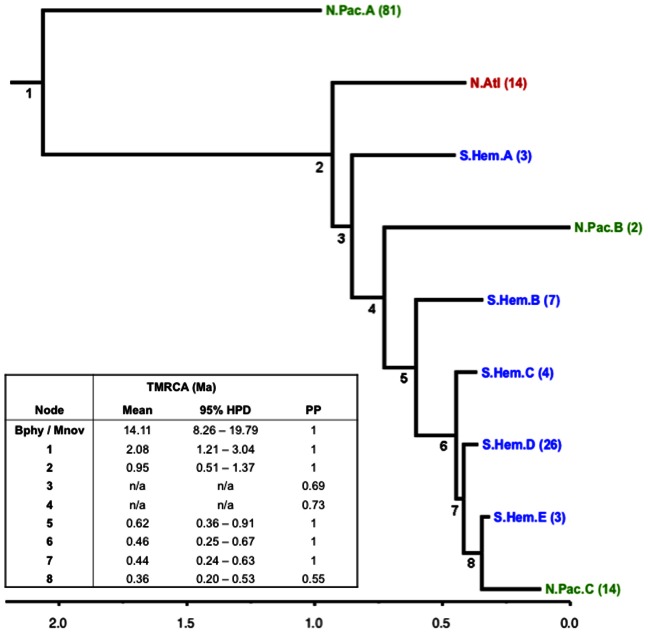
Summary of Bayesian fin whale phylogenetic tree using protein coding mitogenome sequences (*cds* dataset). The root leads to divergence of fin whales from humpbacks. Branches with two or more samples in the same ocean basin have been collapsed. Numbers in parentheses are number of samples at each branch tip. Time to Most Recent Common Ancestor (TMRCA) estimates, 95% Highest Posterior Density (HPD), and posterior probabilities (PP) of each numbered node are given in the inset table. TMRCA values not reported for nodes with PP<0.9. The full annotated tree is available at the Dryad data repository http://dx.doi.org/10.5061/dryad.084g8.

Bayesian estimates of the TMRCA and nucleotide substitution rates from both the full mitogenome (*mito*) and coding region (*cds*) datasets were very similar (Figure 2 and 3). Both analyses produced results with high ESSs and stable, well-mixed posterior samples after approximately 100,000 iterations. Thus, unless otherwise specified, we will only refer to results from the *mito* analysis. Mean substitution rate estimates (2.94e-3, 95% HPD = 1.79e-3–4.45e-3) agree well with estimates from Jackson et al [41] for mysticetes across the mitogenome (3.1e-3, 95% HPD = 2.6e-3–3.7e-3), and Sasaki et al [43] as reported in Nabholz et al [46] between humpback and fin whales (8e-3, 95% CI = 5e-3–2e-2), and between Bryde’s and sei whales (7e-3, 95% CI = 3e-3–1.2e-2) [41], [43], [46]. However our estimates are slightly less than those made by Dornburg et al [45] for within fin whales (1.1e-2, 95% HPD = 9e-3–1.2e-2), and within humpbacks (7.7e-3, 95% HPD = 5.6e-3–9.5e-3) [45].

The Bayesian phylogenetic tree shows a strong association of clades to the three ocean basins (Figure 2 and 3). Most of the North Pacific samples (n = 81) fall within one clade (NP clade A), which diverged from the other fin whales approximately 2 Ma (95% HPD = 1.1–2.8 Ma). All North Atlantic and Mediterranean samples diverged from the remaining Southern Hemisphere and North Pacific samples approximately 1 Ma (95% HPD = 0.6–1.5 Ma). Within this North Atlantic clade, the Mediterranean samples do not share haplotypes with samples from the rest of the North Atlantic, nor do they form a separate clade on their own.

A striking feature in the remainder of the tree is the presence of two clades of North Pacific samples among the Southern Hemisphere samples. The outermost clade (North Pacific clade B) contains only two samples, one from Hawaii and the other from the Gulf of California. The posterior probability (PP) for the node joining this clade to the rest of the samples is relatively low (0.68) in both the *mito* and *cds* trees (0.68 and 0.73 respectively) indicating uncertain placement. This is also evidenced by its position internal to Southern Hemisphere clade A in the *mito* tree and external to it in the *cds* tree. However, the second, more recently diverged clade of 14 North Pacific samples (North Pacific clade C) is well supported (PP = 1) as closely related to Southern Hemisphere samples. This clade is estimated to have diverged approximately 0.37 Ma (95% HPD = 0.19–0.54 Ma). The TMRCA of all samples within this clade is more recent (approximately 0.06 Ma, 95% HPD = 0.06–0.21 Ma).

In order to examine the representation and distribution of North Pacific clades A and C in a larger sample of animals from the North Pacific (ignoring clade B due to its uncertain placement and small sample size), we conducted a simple assignment test of the North Pacific samples for which we had Sanger D-loop sequences that were not included in the NGS mitogenome data. We calculated the mean Jukes-Cantor distance between these samples and all samples in each of the two clades. Samples were then assigned to the clade to which they had the shortest mean pairwise distance.

**Figure 4:**
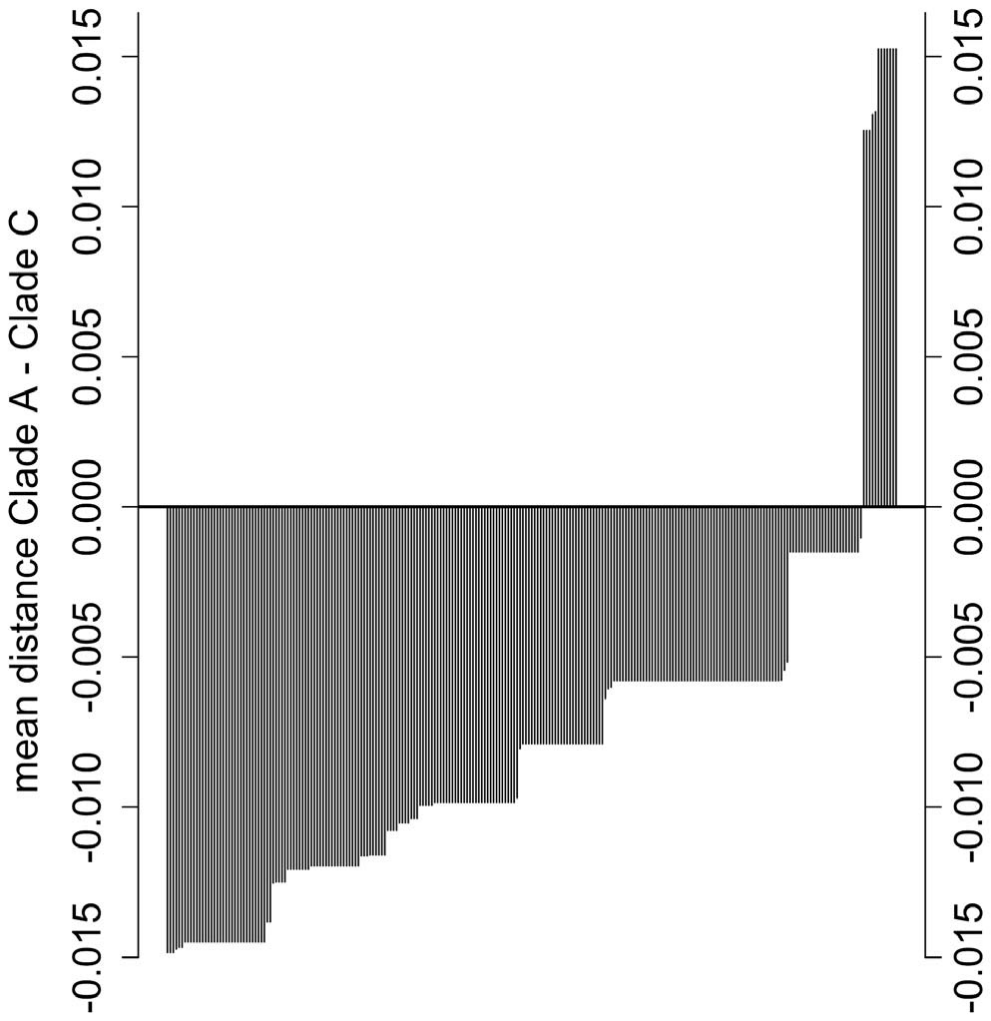
Distribution of difference between mean distances to North Pacific clade A and North Pacific clade C for all mtDNA control region sequences. Values below 0 indicate that the sample is closer to clade A than clade C.

The results of this analysis indicate that although both clades are represented in the larger sample from the North Pacific, there is a slight, but significant difference in the frequencies of geographic strata from which they were sampled. Figure 4 shows the distribution of the difference in mean pairwise distances to North Pacific clade A and C for the 249 North Pacific Sanger-sequenced control region samples. Of these, 237 were closer to the North Pacific clade A, while the remaining 12 were closer to clade C. We then combined these assignments with the 97 NGS samples and examined the frequency distribution of sampling strata by clade (Table 2). Although the overall sample size of clade C is considerably smaller, there is indication that the sampling strata are differentially represented in the clades (χ^2^ *p*-value = 0.014). Most notably, a higher proportion of North Pacific clade A is composed of samples from south of Pt. Conception, California (Gulf of California and Southern California Bight), while a higher proportion of clade C is composed of samples from north of Pt. Conception (California, Oregon, Washington and Gulf of Alaska). Nevertheless, the presence of clade C in all regions examined does not suggest that this clade could represent a relatively isolated group of whales that would warrant separate management as a stock or perhaps subspecies. Samples from both clades A and C were collected together in four out of 15 sampling events where more than one sample was taken. We did not find any differences between the clades in the sex ratio of samples, sampling season, or year.

**Table 2:** Distribution of North Pacific sampling strata in phylogenetic clades from assignment of control region sequences.

Strata	NP Clade A	NP Clade C
**Bering Sea**	21 (0.06)	2 (0.07)
**Gulf of Alaska**	111 (0.35)	12 (0.43)
**California, Oregon, Washington**	38 (0.12)	9 (0.32)
**Southern California Bight**	114 (0.36)	3 (0.11)
**Gulf of California**	32 (0.10)	1 (0.04)
**Hawaii**	2 (0.01)	1 (0.04)

Values are number of samples with the proportion of samples in each stratum in parentheses. Samples include both those assigned from control region sequences and mitogenome sequences used to build the Bayesian tree.

In the full mitogenome there were 27 fixed differences between all North Pacific and North Atlantic haplotypes, and 28 fixed differences between the North Atlantic and Southern Hemisphere (Table 3). Because of the polyphyletic relationship of the North Pacific and Southern Hemisphere, there were no fixed differences between haplotypes from those two ocean basins. However, when North Pacific clades A and C were examined separately, there were 64 and 13 fixed differences between each clade and the Southern Hemisphere, respectively. Interestingly, the two North Pacific clades were more different from one another than either was from the other two ocean basins, with 108 fixed differences between them. The second greatest difference was the 94 fixed differences between North Pacific clade A and the North Atlantic. The number of fixed differences within the more commonly sequenced cytochrome B gene and control region was proportionally similar to the full mitogenome relative to sequence length. The only comparison without at least one diagnostic site aside from the entire North Pacific and Southern Hemisphere was in the control region between North Pacific clade C and the Southern Hemisphere.

**Table 3:** Number of fixed differences between ocean basins and phylogenetic clades in the full mitogenome, cytochrome B, and the control region.

	North Pacific	North Pacific Clade A	North Pacific Clade C	North Atlantic
**Full mitogenome (16,423 bp)**				
North Pacific Clade C	–	108		
North Atlantic	27	94	77	
Southern Hemisphere	0	64	13	28
**Cytochrome B (1,139 bp)**				
North Pacific Clade C	–	11		
North Atlantic	3	11	6	
Southern Hemisphere	0	7	1	2
**Control region (412 bp)**				
North Pacific Clade C	–	1		
North Atlantic	2	3	4	
Southern Hemisphere	0	1	0	1

The first “North Pacific” column represents all North Pacific samples (clades A, B, and C).

## Discussion

The three currently described subspecies of fin whales (*Balaenoptera physalus physalus*, *B. p. quoyi, B. p. patachonica*) have been based on morphological differences found in a limited series of specimens from whaling in the North Atlantic and Southern Ocean [2], [3], [8]. Since then, although there have been genetic and acoustic studies examining population structure within ocean basins, as well as among populations between ocean basins [13], [14], [48], little work has been done to re-examine the taxonomy of this globally-distributed species.

The results of this study, the first to explicitly examine the divergence between North Pacific and North Atlantic fin whales in comparison to the Southern Hemisphere, show strong phylogeographic structuring among these three ocean basins. The North Atlantic (including samples from the Mediterranean Sea) formed the only monophyletic clade, diverging from other fin whales in either the North Pacific or Southern Hemisphere approximately 0.99 Ma (95% HPD = 0.56–1.46 Ma). The polyphyletic North Pacific was distributed in three clades in the tree, the largest of which diverged from all other fin whale haplotypes approximately 1.94 Ma (95% HPD = 1.09–2.81 Ma), and the other two associated with Southern Hemisphere whales. These estimates are congruent with those made by Bérubé et al [14] with North Pacific and North Atlantic/Mediterranean fin whales having diverged 1–3 Ma. These patterns in conjunction with the large number of fixed differences between North Atlantic fin whales and all other ocean basins strongly indicate that *Balaenoptera physalus physalus*, the Northern Hemisphere fin whale, is not composed of a single subspecies.

Among globally distributed mysticetes, like fin whales, the amount of divergence between closely related taxa in each hemisphere and ocean basin varies. It is believed that the generic migratory pattern of large whales (feeding in higher latitudes during the summer months, and travelling to lower latitudes to calve and breed in the winter) serves as a relatively effective barrier to trans-equatorial gene flow [6], [7], leading towards reproductive isolation and ultimately speciation in each hemisphere. However, some species, like blue whales (*Balaenoptera musculus* Linneaus, 1758) can be found near the equator either seasonally or year-round [49], and trans-equatorial migrations and movements between ocean basins have been observed in other species as well [50], [51]. Changes in oceanographic conditions, such as cooling during Pleistocene glacial periods, could force anti-tropical forms closer together, increasing the likelihood of exchange [6]. These processes would be expected to produce a pattern in which anti-tropical pairs with a greater distributional hiatus near the equator are also more taxonomically divergent.

Right whales (*Eubalaena* spp. Gray, 1864), which are distributed in temperate to subpolar waters and are rarely encountered below approximately 15°–20° latitude in either hemisphere [52]–[54], represent one end of this spectrum. For this genus, there is a broad 30°–40° band that separates whales in the two hemispheres. Historically, two antitropical species of right whales were recognized: *Eubalaena glacialis* Müller, 1776 in the Northern Hemisphere, and *E. australis* Desmoulins, 1822 in the Southern Hemisphere [55]. Rosenbaum et al [7] demonstrated that within the mitochondrial control region, *E. glacialis* contained two reciprocally monophyletic matrilines [7]. The matriline containing haplotypes from the North Pacific was more closely related to the Southern Hemisphere *E. australis* than to *E. glacialis* in the North Atlantic, which were basal in the tree. The authors therefore suggested that North Pacific right whales should be elevated to full species status as *E. japonica* Lacépède, 1818, a designation later confirmed with a suite of nuclear loci [56]. Within the 292 bp examined by Rosenbaum et al [7], there were between 6 and 7 fixed differences between *Eubalaena* species. In comparison, in the same region in our fin whale sequences, there are only two fixed differences between North Atlantic and North Pacific samples, one fixed difference between North Atlantic and Southern Hemisphere samples and no fixed differences between North Pacific and Southern Ocean samples.

With a distribution more like that of fin whales, minke whales are represented both by antitropical species and subspecies pairs. The common minke whale (*Balaenoptera acutorostrata* Lacépède, 1804) can be found from subpolar waters during the summer feeding season, to more temperate to tropical waters in the winter breeding period [55], [57]–[59]. Within this species, there are three recognized subspecies: *B. a. acutorostrata* in the north Atlantic, *B. a. scammoni* Deméré, 1986, in the North Pacific, and the dwarf minke (*B. a.* subsp.) in the Southern Hemisphere [55]. The sister species to the common minke whale, the Antarctic minke whale (*B. bonaerensis* Burmeister, 1867) is restricted to the Southern Hemisphere. The TMRCA between the two minke whale species has been estimated to be between 4.4 and 4.9 Ma, and the TMRCA of the three common minke whale (*B. acutorostrata*) subspecies was estimated at 1.2 Ma (95% CI = 0.3–2.2 Ma) [60], very similar to the TMRCA of all fin whale haplotypes in this study (1.9 Ma, 95% HPD = 1.1–2.9 Ma).

The taxonomy of blue whales (*Balaenoptera musculus*), which has yet to be fully elucidated, may represent the other end of this spectrum. In the Pacific, blue whales are found around the productive tropical Costa Rica Dome year round [49], and feeding has been documented off the equatorial Galapagos Islands [61]. It is likely that at least seasonally, waters from south of the Equator to northern Peru can contain blue whales from both the Northern and Southern Hemispheres [49], [62]. The nominate subspecies (*B. m. musculus*) contains whales in the North Atlantic and North Pacific combined. Two additional subspecies are recognized, the subantarctic “pygmy” blue whales (*B. m. brevicauda* Ichihara, 1966), and the Antarctic “true” blue whales (*B. m. intermedia* Burmeister, 1871–72) [55]. Although they are morphologically diagnosable, the amount of genetic differentiation among subspecies described to date is low compared to that found for other recognized subspecies of whales, as well as that found between ocean basins in the fin whale data in this study [62], [63].

Thus, with their temperate distributions, fin whales exhibit patterns of divergence intermediate to those of minke and blue whales. The polyphyletic pattern of North Pacific and Southern Hemisphere haplotypes could represent evidence of introgression between the two oceans. At the very least, it appears that North Pacific clade C (Figure 2) results from a migration event occurring relatively recently, approximately 0.37 Ma. Alternatively, the patterns we see could result from incomplete lineage sorting, reflecting ancestral polymorphism of a much larger population. This would suggest that gene flow between the two ocean basins had ceased earlier, perhaps closer to the 1.94 Ma divergence time of North Pacific clade A. Differentiating between introgression and incomplete lineage sorting is rarely straightforward without other sources of information such as patterns of divergence from nuclear genes [64], [65]. However, given that the divergence of North Pacific clade A is approximately 1.52 Ma older than North Pacific clade C, we believe it is most likely that clade C represents a migration event. The estimated position of North Pacific clade B in the tree, basal to the Southern Hemisphere haplotypes, would be more consistent with incomplete lineage sorting, but the relatively weak PP of this clade (0.64) make any inferences about its origin tenuous at best.

While it is clear that the mitogenome can provide enhanced resolution for phylogenetic patterns [29]–[31], it is nonetheless still inherited as a single locus with multiple linked genes and as such may produce gene trees that are not the same as the “species” trees due to introgression or hybridization [64]. With the rapid growth of Next Generation Sequencing, we are likely to see an order-of-magnitude increase in the number of nuclear loci that can be to be applied to phylogenetic questions [66].

Bérubé et al [13], [14] have shown significant population differentiation between Mediterranean and North Atlantic populations as well as evidence of structure between fin whales from the western and eastern North Atlantic [13], [14]. Acoustic differences between North Atlantic and Mediterranean fin whales have also been described [67]. Although population structure in the North Pacific has not been fully elucidated, Mizroch et al [68] discussed five possible populations, or “feeding aggregations”: the eastern and western groups that move along the Aleutians [69], [70]; the East China Sea group; a group that moves north and south along the west coast of North America between California and the Gulf of Alaska [71]; and the whales in the Sea of Cortez, which have been recognized as a resident population based on both genetic and acoustic differences [13], [15], [72]. Multiple fin whale call types have been described for the eastern North Pacific (personal communication, E. Oleson, Pacific Island Fisheries Science Center, National Marine Fisheries Service) [15], suggesting that there may be further subdivision along the west coast of North America. In light of this, it is intriguing that although we did not see strong phylogeographic structure within ocean basins in our data, we did see differential representation of the North Pacific clades A and C on either side of Pt. Conception, CA. Whether or not population subdivision or diversity in the North Pacific is related to patterns of historical immigration will be better addressed by future analyses of nuclear and acoustic data.

The taxonomic status of North Pacific fin whales is unclear. If analyses of nuclear loci indicate current gene flow between the clade A and clade C mitochondrial matrilines, this would suggest that all eastern North Pacific fin whales are members of a single, new subspecies. On the other hand, if significant differentiation between these two clades is observed in nuclear markers, then further work should be conducted to further describe other differences between these two sympatric forms. Given its placement in the tree, clade C would likely fall within the current definition for *B. p. quoyi*, with the odd result of “Southern Hemisphere” fin whales residing in the North Pacific. The sympatric clade A would then become a new subspecies.

Future genetic studies would be greatly enhanced by the inclusion of more samples from regions in which fin whales are known to occur. All but two of the Southern Hemisphere samples came from a single region sampled over a two-year period [73], [74] and did not encompass the full range of fin whales across the Southern Ocean [75]. The inclusion of samples from the South Atlantic and South Pacific would allow us to clarify the evolutionary relationship of the North Atlantic and North Pacific and help identify potential avenues of dispersal. Additionally, these samples would allow for the examination of further structuring within *B. p. quoyi* in the Southern Hemisphere. In particular, they would be valuable for examining the validity of the low- to mid-latitude pygmy fin whales, *B. p. patachonica*. The description presented by Clarke [8] is primarily based on an examination of whaling records, historical descriptions of external morphology, and the biological examination of one specimen. Thus, genetic samples of whales from this region will be necessary to fully evaluate this proposed subspecies and its relationship to other Southern Hemisphere whales.

In all ocean basins, fin whale populations were greatly reduced by commercial whaling in the early 20^th^ century [68], [76]. As a result, fin whales in both the Atlantic and Pacific are listed as “Endangered” under the United States Endangered Species Act and by the International Union for the Conservation of Nature and Natural Resources (IUCN). In the North Pacific, North Atlantic, and Mediterranean Sea, fin whales appear to be particularly vulnerable to ship strikes [76]–[79]. In the eastern North Pacific, populations are increasing [80]. Their status is uncertain in the North Atlantic and Southern Ocean, regions where limited whaling is still occurring [76]. To effectively manage such a globally distributed species, with variable threats, histories of exploitation, and therefore different levels of recovery in each region, it is important to ensure that the taxonomy appropriately reflects the degree of genetic differentiation and divergence.
